# A Clinical Trial for the Identification of Metabolic Biomarkers in Hashimoto’s Thyroiditis and in Psoriasis: Study Protocol

**DOI:** 10.3390/pathophysiology28020019

**Published:** 2021-06-14

**Authors:** Evangelia Sarandi, Sabine Kruger Krasagakis, Dimitris Tsoukalas, Gottfried Rudofsky, Aristides Tsatsakis

**Affiliations:** 1 Laboratory of Toxicology and Forensic Sciences, Medical School, University of Crete, 71003 Heraklion, Greece; tsatsaka@uoc.gr; 2 Metabolomic Medicine, Health Clinics for Autoimmune and Chronic Diseases, 10674 Athens, Greece; dtsoukalas@einum.org; 3 Dermatology Department, University Hospital of Heraklion, 71500 Heraklion, Greece; krkras@uoc.gr; 4 European Institute of Nutritional Medicine, 00198 Rome, Italy; 5 Clinic of Endocrinology and Metabolic Disorders, Cantonal Hospital Olten, Baslerstrasse 150, 4600 Olten, Switzerland; gottfried.rudofsky@spital.so.ch

**Keywords:** Hashimoto’s, psoriasis, metabolomics, organic acids, fatty acids, metabolic biomarkers, epigenetic factors, quality of life

## Abstract

Hashimoto’s thyroiditis and psoriasis are inflammatory disorders that significantly impact patients’ quality of life, stressing the need for novel biomarkers of early diagnosis. This randomized clinical trial (NCT04693936) aims to identify Hashimoto’s thyroiditis’ and psoriasis’ metabolic biomarkers and to investigate the effect of environmental factors on the disease-related metabolic imprint and quality of life. Patients with Hashimoto’s thyroiditis, patients with psoriasis, and healthy individuals aged 18–60 will be recruited, enrolled according to eligibility criteria (medical history, clinical thyroid markers and the PASI score) and randomized to two groups. The intervention group will receive a combination of nutraceuticals for 6 months as part of a Mediterranean diet, and the control group will follow their usual diet. Data will be collected at baseline and the end of the study, including metabolite levels, lifestyle and anthropometric measurements, adherence to the Mediterranean diet (through the Mediterranean Diet Score) and disease-specific quality of life (through the Thyroid Patient Report Outcome for Hashimoto’s group, and the Dermatology Life Quality Index for the psoriasis group). This study will investigate metabolic biomarkers and related changes in Hashimoto’s thyroiditis and psoriasis and evaluate the association of metabolic changes with dietary factors and quality of life.

## 1. Introduction

Hashimoto’s thyroiditis and psoriasis are two common inflammatory disorders with several complications on overall health and quality of life. Beyond the genetic predisposition, which has been indicated as a risk factor, growing evidence shows that non-genetic triggers, including dietary, lifestyle, and environmental factors contribute to the onset of chronic inflammatory conditions [1].

Hashimoto’s thyroiditis (HT) is an autoimmune disease and the most prevalent thyroid gland disorder affecting 3.5 cases per 1000 subjects annually. Patients with HT have hypothyroidism due to thyroid gland damage, which is caused by an excessive inflammatory response against the thyroid gland, mediated by the auto-antibodies anti-thyroglobulin (anti-TG) and anti-thyroid peroxidase (anti-TPO). Early diagnosis is a crucial determinant to the progression of the disease and the proper management of HT, where it is important to maintain the integrity of the thyroid gland [2]. Beyond the genetic variants that are associated with HT onset, several non-genetic factors have been acknowledged to be important. Vitamin D and selenium levels [3,4], overall dietary habits, and antioxidant intake play a key role in HT pathogenesis through their involvement in critical pathways, including the insulin signaling pathway, redox processes, and gut–thyroid interactions [5,6,7]. Selenium is a trace element with multiple roles on human health and has emerged as a potent preventive and supportive treatment strategy against thyroid diseases. It is required for the biosynthesis of selenoproteins by the thyrocytes, which are essential for the normal function of the thyroid gland, the protection from oxidative damage, and the modulation of the inflammatory response [8]. A recent review analyzing the results from 20 selenium interventional clinical studies concludes that selenium exerts a protective role in autoimmune thyroiditis mediated by the decline of anti-TPO levels. However, validation studies are needed also addressing disease-related quality of life [3]. Vitamin D is a steroid hormone with pleiotropic effects on the immune system and other non-skeletal functions such as improved metabolic health. Specifically for HT, vitamin D levels have been inversely correlated with anti-TPO and thyroid-stimulating hormone (TSH) levels reviewed by Mele et al. [4]. Vitamin D supplementation studies have significantly decreased the risk of developing thyroid disease [9]. In addition, supplementation of HT patients resulted in a significant anti-TPO and anti-TG levels decline, suggesting a beneficial role for the thyroid gland function [10]. The synergistic effects of nutrients on HT were demonstrated through the improved thyroid function markers in HT female patients receiving both selenomethionine and vitamin D compared to those receiving selenomethionine alone [11]. Additional studies are required to explore the potential of nutraceutical supplementation to thyroid function, including also quality of life indexes.

Psoriasis (PSO) is an immune-mediated inflammatory skin disorder that is characterized by an excessive proliferation of keratinocytes and epithelial cells and extensive inflammation leading to red, patchy, itchy, and dry psoriatic skin lesions. PSO is estimated to affect 2–3% of the population globally but has been reported to reach 11% in some countries. An ongoing debate exists regarding the autoimmune nature of PSO since it shares common genetic and biochemical characteristics with other autoimmune diseases and circulating auto-antigen has been identified. However, their involvement in PSO onset has not been elucidated [12]. Environmental factors play a central role in psoriasis onset (e.g., trauma or infection), the disease progression through the aggregation of symptomatology, and the onset of related comorbidities (e.g., psoriatic arthritis, metabolic syndrome, cardiometabolic and gastrointestinal diseases) that deteriorate the quality of life of patients and decrease their life expectancy. Specifically, researchers propose a link between diet-associated obesity and insulin resistance with PSO and a possible role of gut homeostasis [12,13]. Treatment strategies of PSO include topical treatment for mild severity PSO and systemic therapy for moderate and severe PSO. Topical treatment includes corticosteroids; vitamin D analogs; calcineurin inhibitors; keratolytics such as tazarotene, a synthetic retinoid; and anti-inflammatory agents such as fumaric esters. Patients with mild PSO not responding to the topical treatment or having adverse effects are recommended to use oral systemic therapy, either conventional, such as methotrexate and the retinoid synthetic form acitretin, or combined with biologic therapy. Due to the limitation of the long-term use of oral conventional drug treatment, attention has been given to the management of PSO through supplementation with known anti-inflammatory and antioxidant natural source vitamins and compounds, probiotics, and fish oil supplements [14,15,16,17,18,19]. Despite growing evidence on the beneficial role of such nutrients on PSO and related complications, discrepancies of results have stalled their validation and translation to clinical practice, requiring additional and well-designed clinical trials.

Patients with PSO have an increased risk of developing thyroid disease, including HT, and several attempts have been made to unravel the common pathological mechanisms that explain this [20]. From another perspective, HT and PSO share similar characteristics, including chronic low-grade inflammation, long asymptomatic periods that precede the symptoms onset, increased incidence of comorbidity, metabolic disruptions, and links to obesity, insulin resistance, and metabolic syndrome. Therefore, early identification of these risk factors and proper management might contribute to the prevention and alleviation of these conditions. The aim of the present study is to identify disease-related changes in metabolic pathways that are involved in central biological processes in a case-control cohort study employing metabolomics. In addition, supplementation with a combination of nutraceutical supplements will address the impact of dietary modifications on the identified metabolic pathways and the disease-associated quality of life of patients with HT and patients with PSO.

Metabolites are the molecular intermediates of all biochemical reactions, and their analysis through metabolomics permits the evaluation of the physiological function of critical cellular processes. Metabolomics is a part of the -omics sciences and has been increasingly applied in clinical research and biomarker discovery [21]. The inherent advantage of metabolomics is that by measuring the downstream products of the genetic information flow-through, it is directly associated with the phenotype [22]. Significant progress has been made in the field of biomarker discovery using the two types of metabolomics, i.e., untargeted and targeted. A recent review presents the existing yet limited literature of human metabolomics studies in HT and PSO towards the identification of potential metabolic markers [23].

However, up to date, the metabolic imprint of these diseases has not been fully recorded due to the multiple variables that hamper the reproducibility and validation of the findings. In particular, previous metabolic studies in PSO and HT have highlighted possible metabolic markers associated with the disease, but the small number of studies is a critical limiting factor. In addition, differences in the analytical method, the type of sample, the method of quantification, the geographical location of the population, the type and stage of the disease, and the dietary and lifestyle habits of the populations between studies significantly affect the conclusions drawn, hampering the validation of results. An important gap of many metabolomic studies is the absence of repeated measurements at specific time intervals. Metabolite levels are dynamic in response to exogenous stimuli; thus, a longitudinal analysis may deepen our knowledge on the metabolic fluctuations in a specific disease model and empower the biomarker identification methodology [24,25].

In the present study, metabolomics will be used for the quantitative analysis of selected organic and fatty acids (targeted metabolomics), and the metabolic network will be studied in patients with HT and PSO. Metabolic measurement will be performed at two-time points to capture the dynamic metabolite relationships.

To address the impact of non-genetic factors on metabolite levels, we will attempt to identify associations between the metabolic profile of HT and PSO with measurable dietary and lifestyle habits, the disease stage, and the disease-related quality of life. For an in-depth investigation on the role of dietary factors, an interventional plan consisting of specific dietary supplements will be administered in the context of a Mediterranean diet to a sub-group of the study population and their metabolic profile will be monitored. Finally, the impact of the interventional plan on the disease-related quality of life of the patients will be assessed through validated questionnaires for HT and PSO separately.

## 2. Experimental Design

### 2.1. Study Design

This is a parallel randomized interventional study in patients with Hashimoto’s disease, in patients with psoriasis, and in healthy adults. The trial has been registered in ClinicalTrials.gov, where details on the study and a timeline are available (clinicaltrials.gov) NCT04693936. The protocol has been approved by the Research Ethics Committee of the University of Crete (AP 147/10072020). The protocol is in accordance with SPIRIT (standard protocol items: recommendations for interventional trials) guidelines (Appendix A).

### 2.2. Study Population

The study will include healthy adults, patients with Hashimoto’s disease, and patients with psoriasis (a total *n* = 200) that will be recruited in The Health Clinics for Autoimmune and Chronic Diseases centered in Athens, Greece. Primary screening of patients will include a brief medical history by an endocrinologist for Hashimoto’s disease group and a physician for psoriasis and the healthy group. Subjects will be enrolled if they are adults (18–60 years old), male or female, non-obese (BMI < 30 kg/m^2^), and willing to participate in a dietary interventional trial of 6 months. Additionally, for the Hashimoto’s thyroiditis group, clinical thyroid markers will be assessed to determine the disease status and will be categorized accordingly.

Inclusion criteria:

Hashimoto’s disease (HT): The diagnosis of HT was made by clinical findings, presence of thyroid autoantibodies (anti-TPO) in laboratory tests, and gray-scale US findings.
Psoriasis (PSO): The presence of psoriatic lesions and the disease severity will be assessed according to the PASI score.
Healthy group (CO): Non-obese (BMI < 30 kg/m^2^), non-athletes, non-pregnant or lactating women, not been diagnosed with a chronic or acute disease, and not receiving antidepressants, drugs, and supplements. They also need to have normal (TSH) levels or high TSH and the absence of other clinical findings of thyroid malfunction.
Exclusion criteria: malignant or congenital goiter, complete thyroidectomy.

For individuals that do not have recent tests of the thyroid gland, a free-of-charge thyroid function test will be provided, including TSH measurement for healthy adults and TSH, FT3, FT4, anti-TPO, and anti-TG for HT group as per the physician’s instructions.

Eligible patients will be verbally informed on the study design, objectives, and duration as presented in the informed consent. Signed informed consent will be obtained by the study participants. At any time, the participants are willing to withdraw from the study or their disease/overall health deteriorates, their participation in the study will be terminated, and their data will be withdrawn upon the completion of a withdrawal form.

### 2.3. Sample Size Estimation

Sample size estimation in metabolomic studies is usually performed using Bonferroni correction or the Benjamini-Hochberg (BH) method to adjust for multiple comparisons and multiple hypotheses that are simultaneously tested, and the need to adequately controlling the numbers of tests performed is of paramount interest [26,27]. The mathematical presentation, theoretical details, statistical issues, and controversies of these methods are out of the scope of the present work, and the interested reader can find more technical details elsewhere [28]. In the current study, sample size estimation and power analysis were based on Benjamini-Hochberg adjustment given the high number of comparisons and correlations between data, using the MetaboAnalyst (V5.0) software platform [29].

The sample size was calculated based on previously published data (served as “pilot data” for the present analysis) on the identification of significant differences in the level of metabolites of interest [30]. The percentage of missing values was estimated at a level less than 0.3% and was replaced by one-fifth of the minimum positive values of their corresponding variables. In order to minimize technical variability, quantile normalization was undertaken, commonly used in high-dimensional data analysis [31]. We also implemented a cube root transformation, which is a fairly strong transformation for our dataset with an important effect on the shape of our distributions. Lastly, an autoscaling method, based on standard deviation, was applied to analyze the data on the basis of their correlations [32]. Data that were preprocessed indicated that the metrics of interest (t-statistics amongst groups) reasonably followed a near-normal distribution, as expected. Assuming a 5% FDS (a = 0.05), a number of 60 patients was calculated to reach an 80% (β = 1–20%) predicted power.

### 2.4. Intervention

Eligible participants will be randomly divided into the experimental group and the control group (CG). The 1:1 randomization will be performed using an internet platform (https://www.graphpad.com/quickcalcs/index.cfm (accessed on 10 May 2021) for each group (HT, PSO, CO) to ensure a 1:1 allocation ratio per group. The allocation sequence will be concealed until the patient has been recruited in the trial and the collection of baseline data has been made as per the inclusion criteria. Due to the nature of this dietary intervention trial, the intervention will not be blinded from the participants, their families, or the researchers. The experimental group will receive an intervention that consists of a combination of nutraceuticals as part of a Mediterranean diet (NG, nutraceuticals group), while the CG will follow their usual diet. Nutraceutical supplements include: multivitamin, vitamin C, probiotics, omega 3-6-9, calcium-magnesium, and glutamine. Specifically:

1. My Total Health. Multivitamin supplement with 145 elements (vitamins, minerals, amino acids, enzymes, and natural extracts of fruits and vegetables) from the company Meetab Srl (Lumis Research SA) AEO notification number 29398/18-03-2019.
2. My Immuno. Supplement in the form of powder based on ascorbic acid (vitamin C), with the addition of bioflavonoids and amino acids proline and lysine from the company Meetab Srl (Lumis Research SA) AEO notification number 115921/13-11-2018.
3. My Calmag Powder supplement with vitamin D, calcium, and magnesium from Meetab Srl (Lumis Research SA). AEO notification number 123764/04-12-2018.
4. My Omega Krill. Nutritional supplement with balanced amounts of Omega 3, Omega 6 and Omega 9 with the addition of Krill Oil by the company Meetab Srl (Lumis Research SA) AEO notification number 7414/23-1-2019.
5. L-glutamine and Chios Mastiha. Food supplement with high-quality glutamine Kuowa from Japan and Chios mastic from the company Natural Doctor AEO notification number: 58393/27-06-2017.
6. Probiotics 40 Billion. Nutritional Supplement with 40 billion probiotics from 13 live, friendly probiotic strains from the company Natural Doctor AEO notification number: 58393/27-06-2017.

**Figure 1:**
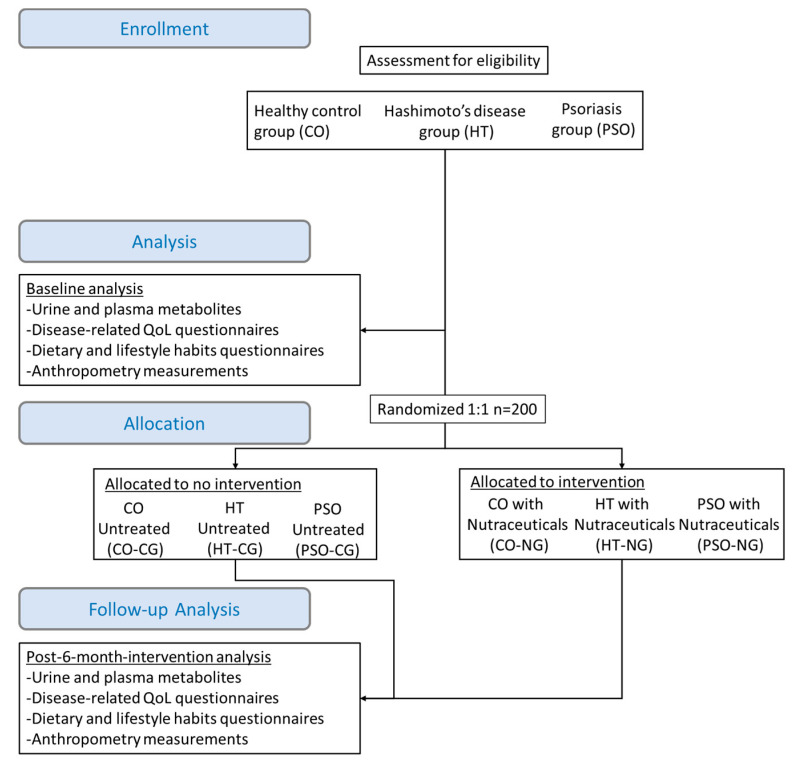
Study flow chart including patients’ enrollment steps, baseline assessment measurements, random allocation to intervention and post-intervention measurements.; QoL: quality of life; NG: nutraceuticals group; CG: control group.

The intervention duration will be 6 months and will include the intake of the abovementioned supplements once a day with no time restriction. The recommendation given will be to take them after breakfast or lunch, but the probiotics to be taken at least 30 min before the first or after the last meal of the day. A flow chart of the study is presented in Figure 1.

### 2.5. Questionnaires

The Mediterranean Diet Score (MDS) will be filled by all participants at baseline and the end of the study to report their dietary habits and adherence to the Mediterranean diet. MDS is a certified questionnaire (PREDIMED) consisting of 14 questions on the frequency and quantity of selected foods. It is a self-administered and close-ended (yes/no) questionnaire that scores the subject from 0–14 depending on the number of products involved in the Mediterranean diet. The score assessment will be as follows: 0–5, lowest adherence; 6–9, moderate adherence; and ≥10–14, highest adherence [33].

The Dermatology Life Quality Index questionnaire (DLQI), a certified quality of life questionnaire specifically for dermatological diseases, will be used by the PSO group to report disease-related changes in the quality of life during the study. Similarly, the HT group will fill the Thyroid Patient Response Outcome (THYPRO) questionnaire, which is certified for the quality of life of patients with thyroid diseases. Patients and study participants being Greek-speaking will fill the Greek validated versions of each questionnaire [34,35].

### 2.6. Metabolomics

For the quantification of organic and fatty acids, urine and peripheral blood samples will be collected from the three groups (HT, PSO, and CO) after fasting and on days 0- and 6 months post-intervention by the nursing staff of the private Health Clinic for Chronic and Autoimmune Diseases.

The method of analysis of selected metabolites will be Gas Chromatography coupled to Mass Spectrometry (GC/MS) based on published methodology. Briefly, 2.5 mL of peripheral blood will be collected from each study participant, and after centrifugation, a total plasma fatty acid analysis will be performed according to Stellaard et al. [36]. Similarly, urine will be collected from the same patients during the day from which organic acids will be isolated and quantified according to the methodology of Tanaka et al. [37].

All statistical analyses of the present work will be conducted using IBM SPSS 22^®^ (IBM Corp., Armonk, NY, USA) software * (https://www.ibm.com/analytics/spss-statistics-software (accessed on 10 May 2021)), a Microsoft Excel^®^ spreadsheet, the free R-project^®^ software (https://www.r-project.org (accessed on 10 May 2021)), and MetaboAnalyst^®^ platform (https://www.metaboanalyst.ca (accessed on 10 May 2021)). Collected data will be anonymized, coded, and stored in an electronic form by the study principal investigator. All data will be checked for administrative typo errors and will be corrected if possible. In the case of missing values due to left censoring (values lower than the limit-of-detection), half of the minimum will be considered as the detection limit, and the rest of the missing values will be replaced by 20% of this value. Nonetheless, if the percentage of missing values overcome 50% of the total observations, the respective variable will be removed. Finally, if the mechanism of missing value is different than mentioned above, several available methods will be considered by the MetaboAnalyst^®^ platform. In order to investigate the assumption that the populations from which the samples are taken are normally distributed, we will use a quantile–quantile plot (the so-called Q–Q plot) in visual checking.

### 2.7. Primary and Secondary Endpoints

Primary Outcome Measure:

1. Differential levels of metabolites quantified by GC-MS between the HT, the PSO, and the CO group to determine baseline disease-related differences and identify potential metabolic biomarkers with predictive value.
2. Change from baseline metabolite levels after the 6-month intervention with nutraceuticals in the HT, the PSO, and the healthy group to determine intervention-related differences on metabolites. HT-NG and PSO-NG will be compared not only to respective CG groups but also to CO-NG, which serves as a positive control. HT-CG and PSO-CG serve as negative controls as they depict the impact of disease progression for the 6-month time course to disease progression or in response to other non-dietary factors.
3. Change from baseline thyroid disease-related and skin disorders-related quality of life at 6 months of intervention with nutraceuticals as assessed by the THYPRO questionnaire and the DLQI questionnaire, respectively. The THYPRO questionnaire will be completed by the HT group and the DLQI by the PSO group at baseline and 6 months post-intervention (both NG and CG). The THYPRO score (0–100) and the DLQI score (0–30) after the intervention will be compared with the baseline score.
4. Change from baseline adherence to the Mediterranean diet at 6 months of intervention with nutraceuticals as assessed by the MDS questionnaire. The MDS questionnaire will be collected for HT, PSO, and control groups (both NG and CG) at baseline and 6 months post-intervention. MDS (0–17) after the intervention will be compared with the baseline score. All of each predictive scores obtained from the THYPRO, DLQI, and MDS could be considered as a Likert-like scale variable and will be analyzed applying parametric analysis since results are comparable with non-parametric tests in most cases [38].

Secondary Outcome Measure:

1. Change from baseline anthropometric measurements at 6 months of intervention with nutraceuticals. BMI will be estimated at baseline and 6 months post-intervention for the three groups (both arms). For the estimation of BMI (kg/m^2^), weight and height will be combined. Waist circumference (cm) will be measured at baseline and 6 months post-intervention for the three groups (both arms).
2. Change from baseline smoking, alcohol consumption, and physical activity at 6 months of intervention with nutraceuticals. Alcohol consumption (number of glasses per week), physical activity frequency (times per week), and smoking (cigars per day) will be assessed at baseline and 6 months post-intervention for all participants (both arms).

Spearman rho correlations for all the combinations amongst the biomarkers will be depicted in a heatmap. The univariate analysis will be performed for all biomarkers based on a Paired Samples *t*-test or the Wilcoxon matched-pairs signed-ranks test (depending on the presence or not of the violation of normality assumption) to compare two measurements (at baseline and 6 months) taken from the same individuals. An independent *t*-test and a Mann–Whitney U-test will also be conducted to compare differences between the means (at baseline and 6 months) for all groups. As mentioned before, the issue of multiple comparisons will be treated within the FDR context, limiting type I error at 5% (a = 0.05). A chi-squared test with continuity correction will also be used to determine whether there is a significant association between gender and the presence of any of these two diseases before and after treatment.

Post-treatment analysis (at the end of 6 months) for the identification of potential predictive biomarkers will be conducted with the use of principal component analysis (PCA) to reduce the number of characteristics to fewer variables that correspond to a linear combination of the originals. In addition, logistic regression will be performed, including the set of the principal components as biomarkers and the presence of or absence of the disease as a dependent variable. Two different models will be developed for PSO and HT. The estimation of the model parameters will be based on the backward selection, which starts with a full model and removes all these variables determined as statistically insignificant. As a sub-analysis, a straightforward logistic regression will be used, including metabolic data and other parameters as independent variables.

As an alternative to non-linear method, we will employ an artificial neural network (ANN) framework to identify biomarkers able to predict the presence or absence of any of these diseases and separate healthy and non-healthy individuals after the treatment. ANN is an established technique in omics and applications in medicine, and related literature can be found elsewhere [39].

Since the structure of any ANN depends on the specific data set at hand, several combinations will be examined to maximize the predictability of the model, limiting the overfitting error. As a general approach, we commend that a Multilayer Perceptron (MLP) feed-forward neural network will be used and trained with the error backpropagation algorithm. A non-strict pre-selection of included variables will be conducted based on the p-value provided by the straightforward logistic regression and/or Spearman’s Rho correlations. To limit further a possible overfitting error, we will limit our hidden layers to two at most. The summary of prediction results will be assessed by the construction of confused matrices and receiver operating characteristic (ROC) curves based on sensitivity and specificity for all the above-mentioned models.

## 3. Discussion

Hashimoto’s disease, the main cause of hypothyroidism, is a major health complication, especially for middle-aged women, as it significantly deteriorates their quality of life and increases the risk of other autoimmune disease incidence. Psoriasis, on the other hand, in addition to the painful and bothering symptomatology, is a common risk factor for psoriatic arthritis and cardiometabolic disorders [40]. Although the mortality rates in these patients are lower compared to other diseases, patients compare their quality of life deterioration to cancer, ischemic disease, and other diseases that are leading causes of death [41]. Diagnosis for HT and PSO is performed based on clinical traits of the disease and only at a stage where the disease is established, stressing the need to discover novel early diagnosis biomarkers. Metabolomics has emerged as a potent tool to identify molecular dysfunctions that indicate the development of a disease pathogenesis years before the symptoms [42]. In this direction, previous studies have focused on the metabolic fingerprint of chronic diseases aiming at the identification of metabolic biomarkers that can discriminate or predict the presence of a disease [30,43,44].

The innovation of the present study includes the quantification of organic and fatty acids in PSO and HT with targeted metabolomics at two time points. The majority of previous metabolomic studies in HT and PSO have employed untargeted metabolomic analysis to identify unexpected changes of the metabolome, thus providing a qualitative evaluation of the metabolic profile of these diseases [23]. The selected metabolic panel of the present study includes metabolites participating in key cellular pathways, including the energy production pathway (Krebs cycle), the detoxification mechanism, and the catabolism of macronutrients carbohydrates, proteins, and lipids, and metabolites related to the microbiome status and vitamin bioavailability. In addition, the present study will include a follow-up assessment of the metabolites of interest to address quantitative changes of metabolites related to disease progression or dietary and lifestyle changes.

In order to capture the effect of diet on the absolute metabolite levels, a validated Mediterranean diet adherence scoring system questionnaire will be used. The Mediterranean Diet Score (MDS) is a self-administered 14-item questionnaire that calculates the level of the participant’s adherence to the Mediterranean diet. MDS has been validated in assessing the association between Mediterranean diet intake and long-term risk of cardiovascular events (CVD) during the PREDIMED study [45]. In addition, it was recently shown that high MDS scores were negatively associated with circulating thyroid hormones, free T3 and free T4, in a healthy overweight/obese population, suggesting the potential implication of Mediterranean diet and thyroid hormone production [46]. With respect to PSO, it was previously demonstrated that low adherence to the Mediterranean diet has a detrimental effect on PSO severity [47], while a later study on psoriatic arthritis demonstrated an inverse correlation between MDS score and disease severity (B = −3.291; 95%CI-5.884 to -0.698) [33]. These encouraging findings indicate a potential association of Mediterranean diet adherence and HT and PSO severity, suggesting that dietary changes may have an impact on their pathogenic mechanisms. Thus, the application of metabolomics will provide a closer look at the effect of the Mediterranean diet on disease pathogenesis by unraveling metabolic changes in response to diet in the context of HT and PSO. Besides, observed changes in metabolites will be adjusted to MDS to unravel the diet-independent correlations of disease-metabolite fluctuations.

An additional strength of the study is the administration of specific dietary supplements in patients with HT and PSO and monitoring of their metabolite fluctuations. Questionnaires specific to thyroid diseases and skin diseases will be given to the participants to provide a better understanding of the relation of identified metabolic changes with the phenotype. The THYPRO is an 85-item questionnaire that captures the quality of life burden of patients with thyroid disease including, tiredness, cognitive impairment, anxiety, goiter, hypothyroidism, hyperthyroidism and eye symptoms, depression, emotional susceptibility, complaints regarding the social and sex life, and overall quality of life [34]. Accordingly, the DLQI is a 10-item questionnaire covering everyday aspects of a patient’s life affected by the skin disease, including pain or disturbance, work, social life and hobbies, exercise, and clothing. Both questionnaires have been suggested that they can be applied in everyday routine clinical practice as they reflect the burden on the patient’s life [35].

Therefore, the present study will address the following: a. Determine the metabolic profile of HT and PSO and identify potential disease biomarkers. b. Investigate the association of metabolic marker fluctuations with dietary, lifestyle, and disease-associated parameters. It should be noted that metabolic biomarkers are by nature very sensitive to environmental factors; thus, the inclusion of these parameters empowers the study design. c. Provide first-level evidence on the effect of a combinatorial nutraceuticals plan on the metabolic markers’ levels and report possible beneficial effects on the disease-specific quality of life of patients. Therefore, two comparisons will be made following the intervention: a. Metabolic profiles of patients with PSO and HT before versus after the intervention. This comparison will also analyze the quality-of-life score before and after the intervention. b. Metabolic profiles in patients with PSO and HT disease after the intervention versus the control group.

The present study protocol has certain limitations that will be cautiously considered during the interpretation of results. The majority of cases with HT and PSO also suffer from concomitant diseases (metabolic syndrome, psoriatic arthritis, dyslipidemia, inflammatory bowel disease, etc.). Patients with comorbidities will be separated from patients with HT only and only PSO so as not to affect the evaluation of the results and will be described in the discussion. In addition, medication will also be reported as a confounder, especially for the PSO group where medication differentiates with disease severity from local to systemic.

The proposed protocol is based on existing literature, however, there are possible problems that have been taken into consideration, such as the insufficient number of participants, where cooperation with an outpatient clinic or hospital will be attempted. In addition, in the case where non-statistically significant differences are found and the inability to find competent biomarkers, additional markers may be looked at. A common issue with cohort interventional studies is the high dropout rate or decreased adherence to the protocol. Thus, the study coordinator will establish telephone communication with the participants to increase the adherence, remind the time of re-examination, assess the dropout rate, and evaluate the need to repeat the enrollment stage to complete the required number of participants.

## 4. Conclusions

Metabolomics is an emerging tool in biomarker discovery with vast application in chronic inflammatory disorders such as HT and PSO. Early identification of metabolic derangements may prove beneficial for disease diagnosis and management, but existing data are scarce. In addition to standard therapeutic strategies for HT and PSO, the role of dietary factors to disease progression, and especially nutraceutical supplements, gains increasing attention. Metabolites are sensitive to environmental changes, thus an efficient tool to monitor the effect of dietary interventions. The present clinical trial will employ metabolomics to identify potential metabolic biomarkers of HT and PSO and study the effect of the combination of nutraceutical supplements to metabolic networks and disease-specific parameters. Overall, the present study will investigate 1. Metabolic markers/pathways that are differentiated in PSO and HT and can be used as diagnostic/prognostic markers 2. Factors affecting the metabolic imprint of PSO and HT 3. Metabolism in HT and PSO in response to dietary intervention and possible association with disease-related quality of life changes.

## Data Availability

Not applicable.

## Appendix A

**Table A1:** SPIRIT 2013 Checklist: Recommended items to address in a clinical trial protocol and related documents *.

Section/Item	Item №	Description	Page Number on Which Item Is Reported
**Administrative information**			
Title	1	Descriptive title identifying the study design, population, interventions, and, if applicable, trial acronym	1
Trial registration	2a	Trial identifier and registry name. If not yet registered, name of intended registry	3
	2b	All items from the World Health Organization Trial Registration Data Set	3
Protocol version	3	Date and version identifier	3
Funding	4	Sources and types of financial, material, and other support	14
Roles and responsibilities	5a	Names, affiliations, and roles of protocol contributors	1,14
	5b	Name and contact information for the trial sponsor	N/A
	5c	Role of study sponsor and funders, if any, in study design; collection, management, analysis, and interpretation of data; writing of the report; and the decision to submit the report for publication, including whether they will have ultimate authority over any of these activities	N/A
	5d	Composition, roles, and responsibilities of the coordinating centre, steering committee, endpoint adjudication committee, data management team, and other individuals or groups overseeing the trial, if applicable (see Item 21a for data monitoring committee)	4
**Introduction**			
Background and rationale	6a	Description of research question and justification for undertaking the trial, including summary of relevant studies (published and unpublished) examining benefits and harms for each intervention	1–3
	6b	Explanation for choice of comparators	1–3
Objectives	7	Specific objectives or hypotheses	4,8
Trial design	8	Description of trial design including type of trial (e.g., parallel group, crossover, factorial, single group), allocation ratio, and framework (e.g., superiority, equivalence, noninferiority, exploratory)	3–4
**Methods: Participants, interventions, and outcomes**			
Study setting	9	Description of study settings (e.g., community clinic, academic hospital) and list of countries where data will be collected. Reference to where list of study sites can be obtained	4
Eligibility criteria	10	Inclusion and exclusion criteria for participants. If applicable, eligibility criteria for study centres and individuals who will perform the interventions (e.g., surgeons, psychotherapists)	4–5
Interventions	11a	Interventions for each group with sufficient detail to allow replication, including how and when they will be administered	4–5
	11b	Criteria for discontinuing or modifying allocated interventions for a given trial participant (e.g., drug dose change in response to harms, participant request, or improving/worsening disease)	5
	11c	Strategies to improve adherence to intervention protocols, and any procedures for monitoring adherence (e.g., drug tablet return, laboratory tests)	10
	11d	Relevant concomitant care and interventions that are permitted or prohibited during the trial	-
Outcomes	12	Primary, secondary, and other outcomes, including the specific measurement variable (e.g., systolic blood pressure), analysis metric (e.g., change from baseline, final value, time to event), method of aggregation (e.g., median, proportion), and time point for each outcome. Explanation of the clinical relevance of chosen efficacy and harm outcomes is strongly recommended	7–10
Participant timeline	13	Time schedule of enrolment, interventions (including any run-ins and washouts), assessments, and visits for participants. A schematic diagram is highly recommended (see Figure)	4–6
Sample size	14	Estimated number of participants needed to achieve study objectives and how it was determined, including clinical and statistical assumptions supporting any sample size calculations	4–5
Recruitment	15	Strategies for achieving adequate participant enrolment to reach target sample size	10
**Methods: Assignment of interventions (for controlled trials)**			
Allocation:			
Sequence generation	16a	Method of generating the allocation sequence (e.g., computer-generated random numbers), and list of any factors for stratification. To reduce predictability of a random sequence, details of any planned restriction (e.g., blocking) should be provided in a separate document that is unavailable to those who enrol participants or assign interventions	5
Allocation concealment mechanism	16b	Mechanism of implementing the allocation sequence (e.g., central telephone; sequentially numbered, opaque, sealed envelopes), describing any steps to conceal the sequence until interventions are assigned	5
Implementation	16c	Who will generate the allocation sequence, who will enrol participants, and who will assign participants to interventions	4,5
Blinding (masking)	17a	Who will be blinded after assignment to interventions (e.g., trial participants, care providers, outcome assessors, data analysts), and how	5
	17b	If blinded, circumstances under which unblinding is permissible, and procedure for revealing a participant’s allocated intervention during the trial	N/A
**Methods: Data collection, management, and analysis**			
Data collection methods	18a	Plans for assessment and collection of outcome, baseline, and other trial data, including any related processes to promote data quality (e.g., duplicate measurements, training of assessors) and a description of study instruments (e.g., questionnaires, laboratory tests) along with their reliability and validity, if known. Reference to where data collection forms can be found, if not in the protocol	6–9
	18b	Plans to promote participant retention and complete follow-up, including list of any outcome data to be collected for participants who discontinue or deviate from intervention protocols	10
Data management	19	Plans for data entry, coding, security, and storage, including any related processes to promote data quality (e.g., double data entry; range checks for data values). Reference to where details of data management procedures can be found, if not in the protocol	4–7,10
Statistical methods	20a	Statistical methods for analysing primary and secondary outcomes. Reference to where other details of the statistical analysis plan can be found, if not in the protocol	7–8
	20b	Methods for any additional analyses (e.g., subgroup and adjusted analyses)	7–8
	20c	Definition of analysis population relating to protocol non-adherence (e.g., as randomised analysis), and any statistical methods to handle missing data (e.g., multiple imputation)	7–8
**Methods: Monitoring**			
Data monitoring	21a	Composition of data monitoring committee (DMC); summary of its role and reporting structure; statement of whether it is independent from the sponsor and competing interests; and reference to where further details about its charter can be found, if not in the protocol. Alternatively, an explanation of why a DMC is not needed	-
	21b	Description of any interim analyses and stopping guidelines, including who will have access to these interim results and make the final decision to terminate the trial	-
Harms	22	Plans for collecting, assessing, reporting, and managing solicited and spontaneously reported adverse events and other unintended effects of trial interventions or trial conduct	4–5
Auditing	23	Frequency and procedures for auditing trial conduct, if any, and whether the process will be independent from investigators and the sponsor	10
**Ethics and dissemination**			
Research ethics approval	24	Plans for seeking research ethics committee/institutional review board (REC/IRB) approval	3,14
Protocol amendments	25	Plans for communicating important protocol modifications (e.g., changes to eligibility criteria, outcomes, analyses) to relevant parties (e.g., investigators, REC/IRBs, trial participants, trial registries, journals, regulators)	4
Consent or assent	26a	Who will obtain informed consent or assent from potential trial participants or authorised surrogates, and how (see Item 32)	4
	26b	Additional consent provisions for collection and use of participant data and biological specimens in ancillary studies, if applicable	-
Confidentiality	27	How personal information about potential and enrolled participants will be collected, shared, and maintained in order to protect confidentiality before, during, and after the trial	
Declaration of interests	28	Financial and other competing interests for principal investigators for the overall trial and each study site	14
Access to data	29	Statement of who will have access to the final trial dataset, and disclosure of contractual agreements that limit such access for investigators	
Ancillary and post-trial care	30	Provisions, if any, for ancillary and post-trial care, and for compensation to those who suffer harm from trial participation	
Dissemination policy	31a	Plans for investigators and sponsor to communicate trial results to participants, healthcare professionals, the public, and other relevant groups (e.g., via publication, reporting in results databases, or other data sharing arrangements), including any publication restrictions	
	31b	Authorship eligibility guidelines and any intended use of professional writers	-
	31c	Plans, if any, for granting public access to the full protocol, participant-level dataset, and statistical code	-
**Appendices**			
Informed consent materials	32	Model consent form and other related documentation given to participants and authorised surrogates	
Biological specimens	33	Plans for collection, laboratory evaluation, and storage of biological specimens for genetic or molecular analysis in the current trial and for future use in ancillary studies, if applicable	

* It is strongly recommended that this checklist be read in conjunction with the SPIRIT 2013 Explanation & Elaboration for important clarification on the items. Amendments to the protocol should be tracked and dated. The SPIRIT checklist is copyrighted by the SPIRIT Group under the Creative Commons “Attribution-NonCommercial-NoDerivs 3.0 Unported” license.
